# Precision cancer monitoring using a novel, fully integrated, microfluidic array partitioning digital PCR platform

**DOI:** 10.1038/s41598-019-55872-7

**Published:** 2019-12-20

**Authors:** Megan E. Dueck, Robert Lin, Andrew Zayac, Steve Gallagher, Alexander K. Chao, Lingxia Jiang, Sammy S. Datwani, Paul Hung, Elliot Stieglitz

**Affiliations:** 1 COMBiNATi Inc, Palo Alto, CA USA; 2 Department of Pediatrics, UCSF Benioff Children’s Hospital, San Francisco, CA USA; 3 UCSF Helen Diller Family Comprehensive Cancer Center, San Francisco, CA USA

**Keywords:** Diagnostic markers, Prognostic markers, Molecular medicine

## Abstract

A novel digital PCR (dPCR) platform combining off-the-shelf reagents, a micro-molded plastic microfluidic consumable with a fully integrated single dPCR instrument was developed to address the needs for routine clinical diagnostics. This new platform offers a simplified workflow that enables: rapid time-to-answer; low potential for cross contamination; minimal sample waste; all within a single integrated instrument. Here we showcase the capability of this fully integrated platform to detect and quantify non-small cell lung carcinoma (NSCLC) rare genetic mutants (EGFR T790M) with precision cell-free DNA (cfDNA) standards. Next, we validated the platform with an established chronic myeloid leukemia (CML) fusion gene (BCR-ABL1) assay down to 0.01% mutant allele frequency to highlight the platform’s utility for precision cancer monitoring. Thirdly, using a juvenile myelomonocytic leukemia (JMML) patient-specific assay we demonstrate the ability to precisely track an individual cancer patient’s response to therapy and show the patient’s achievement of complete molecular remission. These three applications highlight the flexibility and utility of this novel fully integrated dPCR platform that has the potential to transform personalized medicine for cancer recurrence monitoring.

## Introduction

It is becoming increasingly accepted that while Next Generation Sequencing (NGS) is an invaluable tool for broad based sequence characterization, its precision in tracking rare mutant events is low. In a recent study, Stetson *et al*.^1^ indicated that NGS is only accurate in quantification of rare mutant burdens that are >1%. Digital PCR offers clinicians the necessary tool for absolute quantification and enhanced precision tracking of ultra-rare (<1%) mutations.

The concept of Digital Polymerase Chain Reaction (dPCR) was first published by Saiki *et al*. in 1988 highlighting a Poisson distribution of individual PCR target sequences^2^ with Volgelstein *et al*., later coining the term “Digital PCR”^3^. Unlike the popular quantitative PCR (qPCR) where PCR templates are quantified by comparing the rate of PCR amplification of an unknown sample to the rate of a set of known qPCR standards, dPCR has been proven to exhibit higher sensitivity^4^, higher precision^5^ and improved reproducibility^6^. The advent of microfluidics has greatly aided the advancement of dPCR; however, current available partitioning technologies suffer from limitations in three critical areas – low partition volume consistency, low partition number consistency and high dead volume of loaded sample. Droplet-based dPCR technologies^7–9^ have innate issues with droplet-to-droplet volume uniformity that can affect the accuracy of quantification, impact reproducibility and hinder conformance to clinical regulatory requirements. Reagent partitioning that requires user-controlled moving parts or user-applied thin films on the consumable^10–12^ can create non uniformity in partition size. Partition and volume non-uniformity, as well as the potential for instrument contamination will likely be obstacles preventing other dPCR platforms from transitioning from research-use to the clinic. Furthermore, variable partition volumes result in variable distribution of targets inside the partitions, thus undermining the mathematical basis of dPCR and downstream analysis. Fluctuating partition numbers underscore the instability of the partitioning process and introduce unnecessary variables into the assay and limit sensitivity. Unanalyzed sample via a high dead volume in the consumable introduces subsampling error and reduces the overall accuracy of the results. Subsampling error is a function of both the expected target concentration as well as the degree of subsampling. The combination of rare targets with high dead volume synergistically lowers dPCR accuracy and broader based applicability for clinical applications.

Here we present a novel Microfluidic Array Partitioning (MAP) consumable device that utilizes precisely manufactured high volume injection molded arrays combined with precise fluidic control to overcome current dPCR limitations. The high-volume injection molding process ensures that all partitions have a consistent and well-defined sample volume. The partition size is defined by the physical limitation of the microarray wells and not by a stochastic process. The partition array consists of a fixed geometry and ensures a consistent number of partitions for all samples across each experimental run. The number of partitions is defined by the array geometry and is minimally susceptible to the variation in the partitioning process. Coupled with a novel precise fluidic loading process, the MAP consumable device utilizes >95% of input sample and significantly reduces subsampling error compared to other currently available technologies that utilize <80% of the loaded sample. For example, in the BioRad QX200 ddPCR system users routinely only generate between 11,000 and 16,000 analyzable droplets per sample out of a possible 20,000^13^. This means that users are only analyzing between 55% and 80% of their loaded sample.

To address the needs of adopting dPCR as a routine tool for personalized medicine, a complete clinic-friendly single instrument dPCR integrated platform was developed. This dPCR platform was designed for simplified workflows, a reduction in contamination potential and rapid time-to-answer. Using three unique cancer-specific applications we have highlighted the platform’s versatility to perform precision, personalized cancer biomarker monitoring.

## Results

### Novel fully integrated single instrument dPCR platform

**Figure 1:**
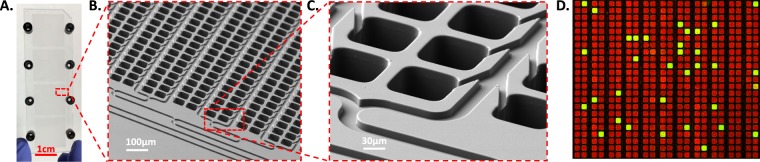
Microfluidic Array Partitioning (MAP) digital PCR (dPCR) consumable. (**A**) Photograph of the MAP consumable. The full micro-molded Cyclo Olefin Polymer (COP) is 1″ × 3″ and contains 4 arrays of 20,000 partitions each. (**B**,**C**) Scanning Electron Micrograph (SEM) images of the MAP consumable partitions. After the automated reagent loading and digitization step, the arrayed partitions (X = 65 µm, Y = 82 µm, Z = 97 µm in height) are filled with reagent and the thinner connecting channels (10 µm in height, 25 µm in width) are next filled with silicone oil (creating fluidically isolated partitions). (**D**) A fluorescent image of the raw dPCR experimental results processed from a MAP consumable. PCR reagents containing a FAM-labeled probe and a reference dye (ROX) were loaded into the consumable and then thermal-cycled per user pre-defined settings. The consumable was next imaged in both the ROX and FAM channels. Finally, these two images were then overlaid where the red (ROX channel) represents partitions in which the reagent successfully loaded but no PCR target was present and the green (FAM channel) represents partitions that contained the PCR target.

**Figure 2:**
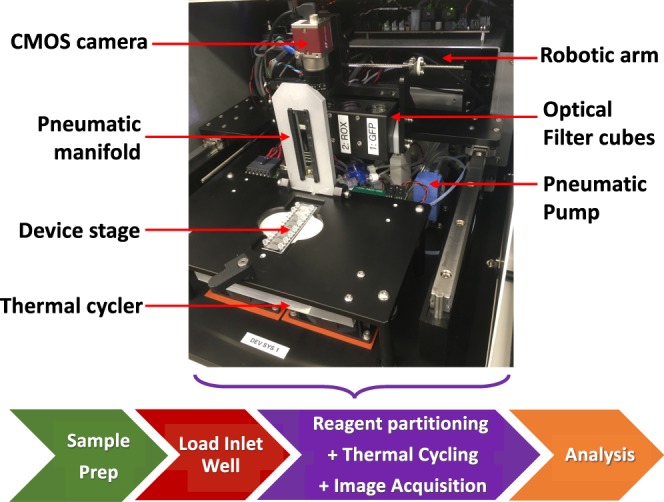
The prototype of the fully-integrated single dPCR instrument. The photographic image shows the various subassemblies and instrument components of the integrated dPCR instrument. The flowchart below represents the simplified workflow where the instrument carries out the reagent partitioning step, followed by the thermal cycling process and finally the results are acquired and software processes the results in a simple one step workflow.

The novel dPCR platform consists of a micro-molded plastic Microfluidic Array Partitioning (MAP) consumable device (Fig. 1) and a fully integrated single instrument that combines pneumatic consumable sample loading, thermal cycling, 3-color fluorescence image acquisition with control and analysis software (Fig. 2, Supplemental Fig. 1). The combination of the MAP consumable and fully integrated single instrument creates a streamlined workflow, reduces the potential for contamination, minimizes human handling errors and has a unique capability to decrease the time to result. The low thermal mass of each microchamber (<1 ng) allows for fast dPCR thermal cycling with reduced dwell times. Since the micro-molded dPCR partitions are shallow (X = 65 µm, Y = 82 µm, Z = 97 µm in height) and located directly above the thermal-cycling heating element (separated only by a 40 µm thin film from STRATEC Inc., Anif, Austria), sample results can be obtained using <15% of the standard thermal cycling dwell times as compared to standard tube-based thermal cycling of droplet based dPCR aggregates (Supplemental Fig. 2). 95 °C PCR enzyme activation can be achieved in 60 seconds (10 minutes recommended), and complete thermal-cycling can be achieved with 40 cycles of 60 °C for 15 seconds followed by 95 °C for 4 seconds. Low thermal mass is an advantage in a system such as this as it reduces the reaction time from the input of energy to deliver a change in temperature, thus enabling better control, as previously demonstrated by Amasia *et al*.^14^ and Farrar *et al*.^15^. The combination of active heating and low thermal mass synergistically enables faster rates of temperature ramp while still maintaining uniform temperature profiles across the MAP consumable. These combined features allow this novel platform to achieve sample-to-answer in <90 minutes with no change to reagent composition.

The MAP consumable partitions each sample into 20,000 micromolded array wells without the need for valves, user-applied films or any moving parts. After the user pipettes 10.5 µL of bulk PCR reagent into a MAP consumable inlet, the user next overlays 5 µL of silicone oil into the same inlet well. After these two liquid handling steps (which can be automated), the inlet wells are capped (and hence closed off) with specialized caps that allow for positive pressure to pass through. The consumable is then loaded into the instrument where the automated loading protocol is initiated. Figure 2 shows a photograph of the interior of the integrated platform as well as a workflow chart indicating the sample loading and analysis steps. Additional details on the construction of the instrument as well as the optical set-up can be found in the supplement (Supplemental Fig. 1).

**Figure 3:**
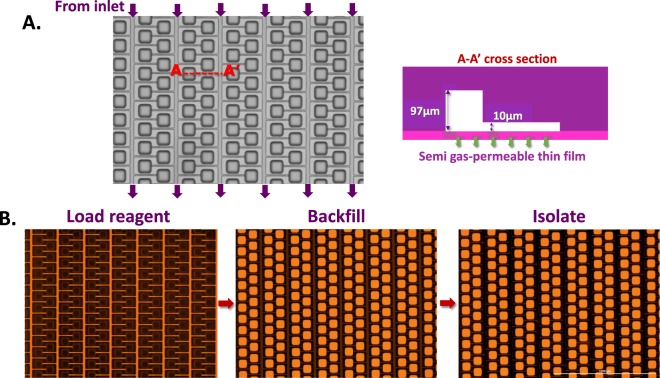
Principle of digitization in MAP consumable. (**A**) MAP consumable brightfield microscopy image accompanied by a cross-section depiction of channel heights. (**B**) The MAP consumable is loaded by applying positive pressure to an inlet well that has been loaded with reagent then overlaid with silicone oil. First the reagent (fluorescent orange) enters the connecting channels adjacent to the partitions. Next the reagent dead-end fills the partitions by outgasing air through the semi gas-permeable thin film. Finally the silicone oil (not visible) enters the connecting channel and fluidically isolates each partition as it passes.

The mechanism by which the MAP consumable is loaded relies upon the 40 µm thin Cyclo Olefin Polymer (COP) film to which the micromolded features are bonded. When positive pressure is applied to a reagent-containing inlet well this 40 µm thin film becomes gas permeable. As the reagent enters the microfluidic features, air is outgassed through the thin film. This allows reagent to completely fill dead-ended features such as the dPCR partitions and also removes all bubbles from inside of the microfluidic features (Fig. 3). First the reagent enters the shallow connecting channels adjacent to the partitions, next the reagent enters and fills the partitions, then oil that was overlaid on the reagent in the inlet well enters the connecting channel and successively fluidically isolates each partition that it passes. Complete filling of partitions was verified using high resolution fluorescence microscopy (Fig. 3). Partition loading success (% of partitions fully loaded) was found to be >98%. Using a 3D surface profiler (Keyence; Itasca, IL) each partition was found to be 65 µm × 82 µm × 97 µm with a volume of 517 pL. Thus, the total volume of reagent in the 20,000 microwell array after digitization is 10.34 µL. Given our partition fill success rate, this means that over 95% of the 10.5 µL of reagent loaded into the inlet well is analyzed in our partition array. Positive pressure directed into the consumable during the full PCR process ensures new bubbles will not form.

The loading of the MAP consumable and the reagent partitioning is accomplished in one instrument by applying positive air pressure to the inlet well with a pneumatic pump (Parker Instrument, standard diaphragm pump; Huntsville, AL). The cap applied on the inlet well prevents the instrument from contacting any potentially contaminated surfaces. Positive displacement pressure of 50 psi directed into the MAP consumable throughout the workflow ensures that no aerosolized contaminants will enter the instrument. This novel fully integrated instrument is dry, contamination-free and requires no routine maintenance to allow for the potential to transition to routine clinical use.

Fully automated software controls the sample digitization into the MAP partitions, thermal cycling and imaging. The partition arrays are imaged before and after PCR thermal cycling in each of the fluorescent dye colors enabling the analysis software to subtract the background fluorescence and correct for non-uniform excitation. A ROX quality control dye that is added to the PCR reaction mix is visible in all partitions and used to confirm the sample was loaded completely and to locate each partition. Partitions without a ROX signal are rejected to reduce the number of false negatives. For each PCR probe dye color, partitions with a significant increase in brightness after PCR thermal cycling are considered positive. The ratio of positive partitions relative to the total number of partitions is used to calculate the sample concentration by applying a Poisson distribution analysis. To initially verify the capabilities of our system, pre-quantified standards from a Next Generation Sequencing (NGS) library quantification kit were run (Takara Cat No. 638324). Quantitative results from the novel platform showed great linearity across dilutions and also small coefficients of variation across replicates (Supplemental Fig. 3).

### EGFR T790M rare mutant quantification

**Figure 4:**
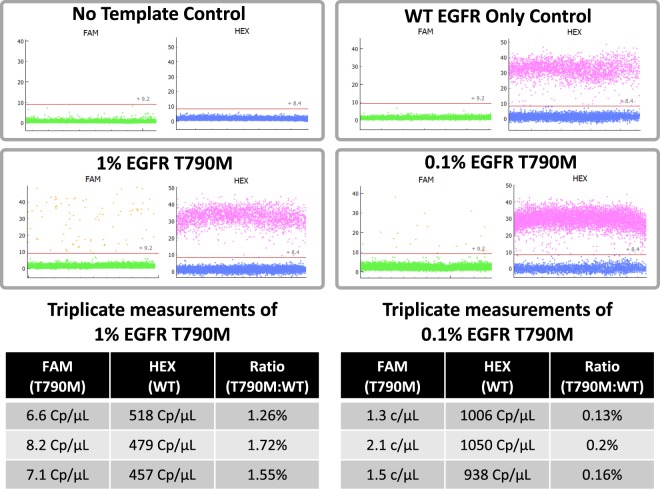
EGFR T790M rare mutant quantification. The reagent was prepared to contain either no template, WT EGFR only, 1% EGFR T790M in a background of WT EGFR or 0.1% EGFR T790M in a background of WT EGFR. The reagent also contained a FAM-labeled EGFR T790M probe along with a HEX-labeled WT EGFR labeled probe. This reagent was loaded into a MAP consumable and processed on the prototype integrated dPCR instrument. A scatter plot of the results show the individual partition fluorescence on the y-axis and partition number on the x-axis (where each data point represents the results from an individual partition). The two tables show the results from measurements in triplicate for the 1.0% and 0.1% EGFR T790M samples.

EGFR is an important drug target for the treatment of non-small cell lung carcinoma (NSCLC). During the treatment of NSCLC with tyrosine kinase inhibitors (TKIs), there is typically an early response, followed by relapse upon outgrowth of a pre-existing subclonal mutation or the acquisition of a de-novo mutation that confers resistance. Early detection of resistance mutations can better inform patient treatment and guide appropriate more efficacious drug selection. One key EGFR mutation that leads to TKI resistance is the T790M mutation. There are only a few clinical assays that have been approved as companion diagnostics in patient biopsies (FFPE or plasma), while a larger number of laboratory developed tests (LDTs) under CLIA/CAP guidance are finding routine use in cancer disease diagnosis or cancer treatment monitoring. EGFR T790M mock cell-free DNA (cfDNA) standards (SeraCare, SeraSeq ctDNA) in combination with a commercially available EGFR T790M dPCR assay (Bio-Rad, ddPCR assay) were used to validate the novel platform presented in this study. The cfDNA standards tested were synthetically manufactured, fragmented and then blended with well-characterized background material to fully mimic cfDNA that would be purified from a patient blood sample. Premixed PCR reagent containing mastermix, the EGFR T790M dPCR assay and the following cfDNA standards were run in triplicate units on the platform: 100% wild-type(WT) EGFR, 1.0% EGFR T790M and 0.1% EGFR T790M (Fig. 4). All replicates for the WT EGFR only control showed no positive partitions in the T790M (FAM) channel showing that the probe was specific to the WT amplicon and that there was no optical crosstalk into the FAM channel. Thresholds for positives in both the FAM and HEX channels were set using the no template control. For the 0.1% and 1% EGFR T790M samples, concentrations were generated for both WT EGFR and EGFR T790M. For example, in one of the triplicate runs using the 0.1% T790M sample, the concentration of WT EGFR derived by the platform was 1006 C_p_/µL and 1.3 C_p_/µL for EGFR T790M. Therefore, the fraction of EGFR T790M: WT for this run was determined to be 0.13%. The Seraseq synthetic cfDNA standards are generated to be fragmented in a manner similar to human cfDNA. As such, small discrepancies between the nominal values supplied by the vendor (e.g.: 0.1%) and those achieved on the novel dPCR platform (e.g.: 0.13%) could be explained by the fragmentation process used to generate the synthetic samples. These results show the novel dPCR platform’s capability to precisely quantify samples containing <1.0% T790M EGFR in a background of WT EGFR with high reproducibility, high accuracy and high specificity.

### BCR-ABL1 rare transcript quantification

**Figure 5:**
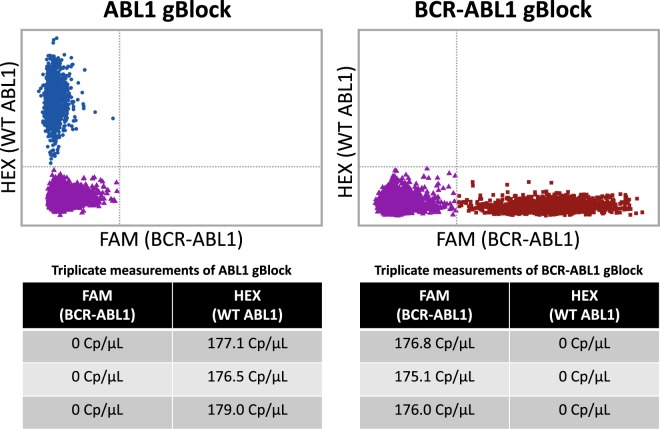
BCR-ABL1 and ABL1 synthetic gBlock nominal concentration determination. The reagent was prepared to contain either ABL1 gBlock solution as template or BCR-ABL1 gBlock as template. The reagent also contained both a FAM-labeled BCR-ABL1 probe in addition to a HEX-labeled ABL1 probe. This reagent was then loaded into a MAP consumable and an experimental run was carried out on the prototype dPCR instrument. A scatter plot of the results show HEX fluorescence on the y-axis and FAM fluorescence on the x-axis (where each data point represents the results from an individual partition). The two tables contain the averaged results of the measurements in triplicate for the individual gBlock templates.

**Figure 6:**
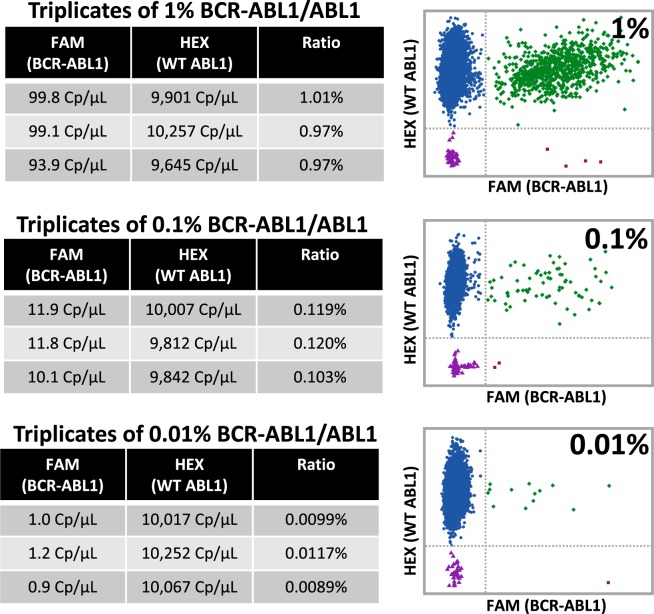
Quantification of precise BCR-ABL1/ABL1 ratios. Using the nominal gBlock concentrations determined prior, precise combinations of gBlock were generated to contain either 1%, 0.1% or 0.01% BCR-ABL1 gBlock in a background of ABL1 gBlock. The reagent was prepared to contain one of these combinations as the template. The reagent also contained both a FAM-labeled BCR-ABL1 probe in addition to a HEX-labeled ABL1 probe. This reagent was loaded into a MAP consumable and run on the prototype dPCR instrument. A scatter plot of the experimental results show HEX fluorescence on the y-axis and FAM fluorescence on the x-axis (where each data point represents the experimental results from an individual partition). The three tables show averaged results from measurements in triplicate for the individual gBlock combinations: 1%, 0.1% and 0.01% BCR-ABL1.

Nearly 95% of chronic myeloid leukemia (CML) cases are characterized by the presence of the BCR-ABL1 fusion gene. Wild type (WT) ABL1 is a tyrosine kinase that plays a role in cell cycle regulation and cellular differentiation. The expression of the BCR-ABL1 fusion oncogene generates a constitutively active tyrosine kinase leading to uncontrolled cellular proliferation and eventually cancer. The advent of tyrosine kinase inhibitor (TKI) therapy has had a transformative effect on CML patient outcomes. Regular monitoring involving precise quantification of BCR-ABL1 transcripts is integral to successful TKI treatment, occasionally leading to drug discontinuation upon achievement of deep remissions. The current gold standard for clinical BCR-ABL1 transcript quantification is Reverse Transcription Quantitative Polymerase Chain Reaction (RT-qPCR). A well-characterized single-plex RT-qPCR BCR-ABL1 assay was previously modified by Alikian *et al*.^16^ to a duplex dPCR assay. Using this dPCR assay, synthetic gBlocks (double stranded DNA fragments) for both ABL1 and BCR-ABL1 were quantified to determine their nominal concentrations (Fig. 4). These synthetic gBlock samples were quantified on the dPCR platform using a FAM-labeled BCR-ABL1 probe and HEX-labeled ABL1 probe. Running the gBlock templates separately showcases the lack of optical cross-talk using the dPCR platform: when pure WT ABL1 gBlock is run there are no positive partitions in the FAM channel, when pure BCR-ABL1 gBlock is run there are no positive partitions in the HEX channel (Fig. 5). To showcase the linearity of our results, a 10X dilution series of BCR-ABL1 gBlock was also run on the system (Supplemental Fig. 4). Thresholds for positives in both the FAM and HEX channels were set using the no template control for all samples. These experimentally derived nominal gBlock concentrations were then used to generate samples with precise ratios of synthetic BCR-ABL1 to ABL1 which were then run on the novel dPCR platform (Fig. 6). For example, in one of the triplicate experimental runs using a 0.01% BCR-ABL1/ABL1 sample, the concentration of WT ABL1 derived by the platform was 10,252 C_p_/µL and 1.2 C_p_/µL for BCR-ABL1. Thus, the fraction of BCR-ABL1/ABL1 for this run was determined to be 0.0117%. Replicate measurements for this same sample were found to be 0.0099% and 0.0089% indicating high reproducibility and high precision. This showcases the platform’s ability to precisely quantify BCR-ABL1 transcripts down to a 0.01% mutant allele frequency with high reproducibility across triplicate runs.

### Personalized cancer monitoring using a patient-specific dPCR assay

To demonstrate the platform’s potential utility in personalized medicine, a droplet digital PCR (ddPCR) assay that was previously designed for precision monitoring of a specific patient was replicated on the novel digital PCR platform. Juvenile myelomonocytic leukemia (JMML) is an aggressive myeloproliferative neoplasm of childhood with nearly half of all patients relapsing within three years^17,18^. A seven-week-old boy diagnosed with JMML was shown to have WT status for all known JMML mutations. Both an institutional DNASeq panel and RNASeq revealed an inframe *CCDC88C-FLT3* fusion^19^. Prior to this discovery, *FLT3* fusions had not been reported in any pediatric malignancy. Given the novelty of this *CCDC88C*-*FLT3* fusion, no commercial assays were available to identify fusion transcripts below the sensitivity of cytogenetics. As such, the oncologist designed a specific ddPCR assay to analyze the abundance of this *CCDC88C*-*FLT3* fusion in peripheral blood RNA over serial time points^18^. This ddPCR-based method for monitoring the patient’s response to Sorafenib (Nexavar) enabled the patient to proceed safely to hematopoietic stem cell transplantation (HSCT) and eventually achieve a complete molecular remission.

**Figure 7:**
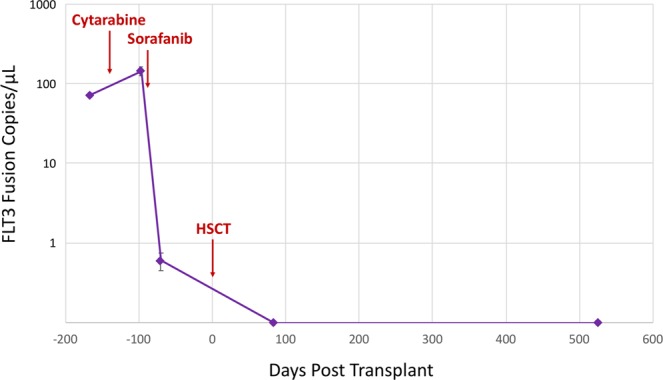
Personalized dPCR tracking of JMML patient’s complete molecular remission. Biobanked cDNA samples from an individual JMML patient were processed on the prototype dPCR integrated instrument using a *FLT3* fusion gene assay designed specifically for this patient. Equal amounts of patient cDNA were used as template for each experimental run. The experimental results indicate that the patient did not respond to the Cytarabine treatment, however the patient showed a dramatic response to Sorafanib which allowed the patient to proceed safely to a hematopoietic stem cell transplant (HSCT). After HSCT all dPCR measurements were true zeros for *FLT3* fusion copies/μL showing that the patient had achieved a complete molecular remission.

Replicating this assay on the prototype dPCR platform with bio-banked samples from the same patient revealed that the fusion transcript levels in the patient’s peripheral blood did not decrease following cytotoxic chemotherapy but then dropped following Sorafenib monotherapy and reached undetectable levels following HSCT (Fig. 7). Thresholds for *CCDC88C*-*FLT3* fusion positive dPCR partitions were set using the no template control. All measurements post-HSCT were true negatives on the novel dPCR platform, showing zero positive partitions containing the *CCDC88C*-*FLT3* fusion target. These results replicated the trends achieved with this assay and samples from this patient using a commercially available ddPCR platform^19^ indicating a high level of concordance. This personalized medicine approach to precision monitoring of patient-specific biomarkers showcases the potential for dPCR as a powerful tool for companion diagnostics.

## Discussion

Here we have presented a novel fully integrated digital PCR platform that was designed to facilitate the adoption of dPCR for precision oncology-based diagnostics. We have showcased the platform’s utility for quantifying <1.0% mutant burden using two clinically relevant applications (EGFR T790M and BCR-ABL1). Furthermore, we demonstrated the utility of this platform for a personalized medicine application by replicating a published clinical study that tracked a patient’s transition to complete molecular remission using a custom, patient-tailored dPCR assay.

Finally, the MAP consumable and fully integrated single system dPCR platform presented is the first of its kind that has potential to allow for real-time and melt-based analysis of digital PCR partitions. Melt-based multiplexing is the key to unlocking the potential for dPCR to produce higher plex quantitative assays, allowing clinicians to precisely quantify panels of 10 or more nucleic acid biomarkers per sample run. In fact, it has previously been shown that a computer can be trained to recognize 89 unique, amplicon-specific PCR melt-signatures^20^. Future integration of our dPCR platform with artificial intelligence (AI) and machine learning could enable unparalleled dPCR multiplexing capabilities.  Real-time monitoring is also critical for improving assay development and can be used to improve partition classification over traditional endpoint dPCR alone. Real time measurement of dPCR partitions can potentially remove the need for arbitrary thresholding entirely. These features combined with dPCR’s innate ability to quantify ultra-rare (<1.0%) genetic events will unlock the power of dPCR for precision cancer monitoring and for improving patient outcomes to advance human health.

## Methods

### MAP consumable fabrication

MAP consumables were micro-injection molded with high optical clarity Cyclo-Olefin Polymer (COP) (Zeon chemical) and sealed with a thermoplastic thin film. Using con-focal laser interferometry, the devices have shown dimensional variability within ±0.1 µm in planar and ±1.0 µm in height, which translated to ±1.5% total volume variation. In addition, a lamination strength study was conducted to show that the devices could withstand over 100 Psi pressure without delamination for over 24 hours.

### EGFR T790M rare mutant quantification

Seraseq® ctDNA EGFR T790M reference standards with nominal (company provided) values were acquired from SeraCare (Seraseq ctDNA EGFR T790M MM AF0.1% Cat. 0710–0707, Seraseq ctDNA EGFR T790M MM AF1% Cat. 0710–0706, Seraseq ctDNA MM WT Cat. 0710–0705; Milford, MA). These reference standards were used as the template in dPCR reactions containing a commercially available EGFR T790M dPCRassay (Bio-Rad ddPCR Assay EGFR T790M Cat. 1016323 and Bio-Rad ddPCRAssay EGFR WT for T790 Cat. 1016326; Hercules, CA). The probe for EGFR WT fluoresces in the HEX channel, whereas the probe for T790M fluoresces in the FAM channel. Primers, probes and Seraseq template were combined with the following dPCR master mix and ROX loading control dye: QuantStudio 3D Digital PCR Master Mix v2 (ThermoFisher Cat. A26358; Waltham, MA), ROX Reference Dye (Biotium Cat. #29052; Fremont, CA). Once mixed, 10.5 µL of the complete dPCR reagent was loaded into an inlet well on the consumable and overlaid with 5 µL of silicone oil (Sigma Cat. 378321; St. Louis, MO). In the final reagent mix the mastermix was diluted 2X, the ROX dye 100X, the primer/probe assay mix 20X, the template 10X, then brought up to volume with water. After the instrument had completed the reagent digitization, the consumable was thermal-cycled using the following program: 1 cycle of 95 °C for 1 minute, 40 cycles of 95 °C for 4 seconds followed by 60 °C for 18 seconds. After running this program the instrument captured images of the various partition arrays using 3 filter sets: ROX, FAM and HEX.

### BCR-ABL1 rare transcript quantification

Primer, probe and full amplicon sequences for both the BCR-ABL1 and ABL1 dPCR targets were derived from the study by Alikian *et al*. (2017). Primers, probes and full BCR-ABL1 and ABL1 template sequences (as synthetic double stranded DNA gBlocks) were custom ordered from Integrated DNA Technologies (Coralville, IA). The probe for ABL1 fluoresces in the HEX channel, whereas the probe for BCR-ABL1 fluoresces in the FAM channel. Primers, probes and gBlock template were combined with the following dPCR master mix and ROX loading control dye: QuantStudio 3D Digital PCR Master Mix v2 (ThermoFisher Cat. A26358; Waltham, MA), ROX Reference Dye (Biotium Cat. #29052; Fremont, CA). In the final reagent mix the mastermix was diluted 2X, the ROX dye 100X, the primer/probe assay mix 20X, the template 10X, then brought up to volume with water. Once mixed, 10.5 µL of the complete dPCR reagent was loaded into an inlet well on the consumable and overlaid with 5 µL of silicone oil (Sigma Cat. 378321; St. Louis, MO). After the instrument had completed the reagent digitization, the consumable was thermal-cycled using the following program: 1 cycle of 95 °C for 1 minute, 40 cycles of 95 °C for 4 seconds followed by 60 °C for 18 seconds.. After running this program the instrument captured images of the various partition arrays using 3 filter sets: ROX, FAM and HEX.

### Personalized cancer tracking using patient-specific dPCR assay

Primers and FAM-labeled probes were designed to amplify and detect CCDC88C-FLT3 fusion cDNA templates according to the methods described by Chao *et al*. (2019). RNA was extracted from peripheral blood and bone marrow using the RNeasy kit (Qiagen Cat. 74104, Hilden, Germany) and 100 ng RNA was reverse transcribed using the Maxima H Minus RT kit (ThermoFisher Cat. K1681; Waltham, MA) and used as a template for dPCR. To prepare the dPCR reactions, a mastermix was prepared using equivalent to 10 ng RNA of cDNA per 10.5 µL reaction. Primers, probes and cDNA template were combined with the following dPCR master mix and ROX loading control dye: QuantStudio 3D Digital PCR Master Mix v2 (ThermoFisher Cat. A26358; Waltham, MA), ROX Reference Dye (Biotium Cat. #29052; Fremont, CA). In the final reagent mix the mastermix was diluted 2X, the ROX dye 100X, the primer/probe assay mix 20X, the template 10X, then brought up to volume with water. Once mixed, 10.5 µL of the complete dPCR reagent was loaded into an inlet well on the consumable and overlaid with 5 µL of silicone oil (Sigma Cat. 378321, St. Louis, MO). After the instrument has completed the reagent digitization, the consumable was thermal-cycled using the following program: 1 cycle of 95 °C for 1 minute, 40 cycles of 95 °C for 4 seconds followed by 60 °C for 18 seconds. After running this program the instrument captured images of the various partition arrays using 2 filter sets: ROX and FAM.

## Supplementary information

Supplementary Information

## Data Availability

The datasets generated or analysed during the current study are available from the corresponding author on reasonable request.
