# Supplementary material for: Precision cancer monitoring using a novel, fully integrated, microfluidic array partitioning digital PCR platform
Source: Sci Rep. 2019 Dec 20;9:19606. doi: 10.1038/s41598-019-55872-7 (PMC6925289; doi:10.1038/s41598-019-55872-7)
Supplement: Supplementary file 1 — Supplementary Information [file 41598_2019_55872_MOESM1_ESM.pdf]

SUPPLEMENTARY INFORMATION

Precision cancer monitoring using a novel, fully integrated, microfluidic array partitioning digital PCR platform

Megan E. Dueck[1*], Robert Lin[1], Andrew Zayac[1], Steve Gallagher[1], Alexander Chao[2], Lingxia Jiang[1], Sammy S. Datwani[1], Paul Hung[1] and Elliot Stieglitz[2,3]

[1]COMBiNATi Inc. Palo Alto, CA.

[2]Department of Pediatrics, UCSF Benioff Children's Hospital, San Francisco, CA.

[3]UCSF Helen Diller Family Comprehensive Cancer Center, San Francisco, CA.

*Corresponding author: megan@combinati.com

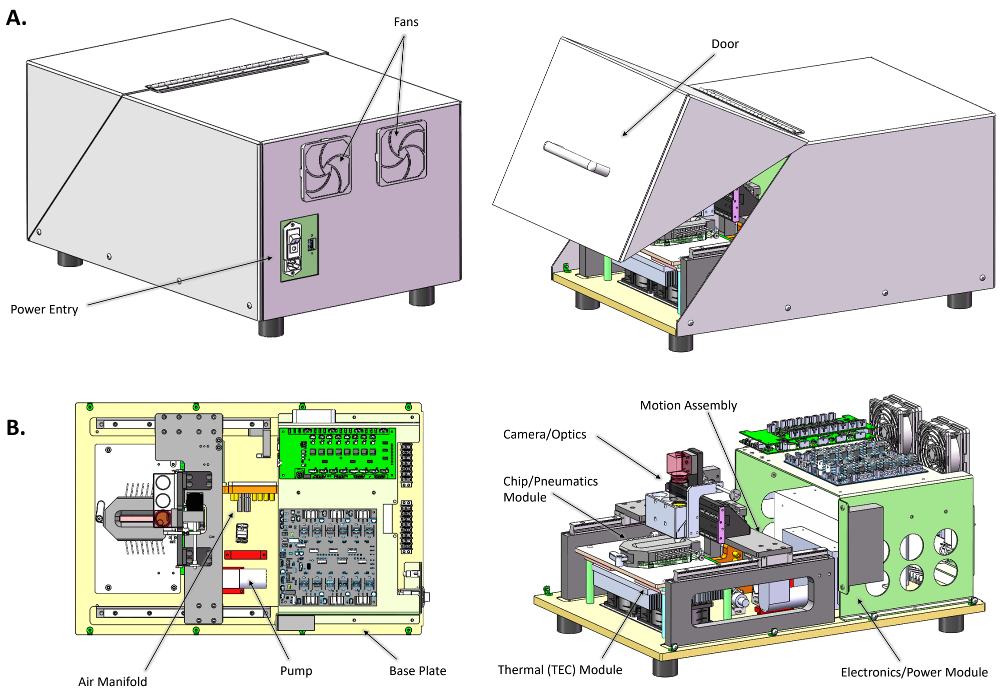

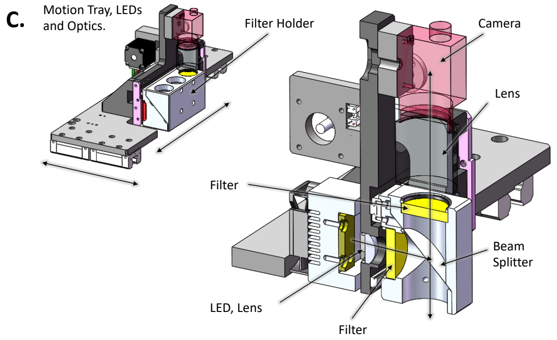

Channel	Excitation	Emission
FAM	470 nm	525 nm
HEX	530 nm	575 nm
ROX	575 nm	623 nm

Supplemental Figure 1: *Detailed schematics of instrument and optical set-up.* A) Broad overview of exterior instrument components. B) Detailed CAD drawings of all major interior instrument components. C) Detailed schematics of the optical set-up within the instrument along with excitation and emission wavelengths for the 3 optical channels. Additional details on specific parts and part numbers can be supplied upon request to the corresponding author. Renderings were generated by Accel Biotech (Los Gatos, California). COMBiNATi holds the rights to these images through a development agreement with Accel Biotech.

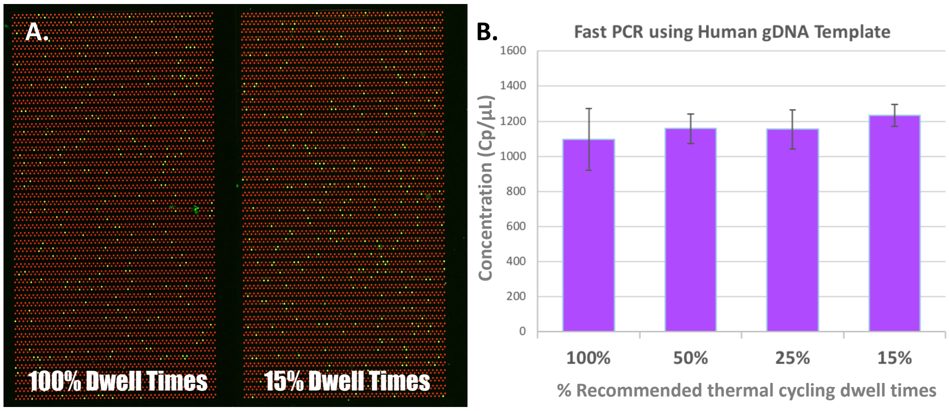

Supplemental Figure 2: *Fast dPCR utilizing the MAP consumable integrated with the single dPCR instrument.* A significant reduction in the thermal cycling dwell time is facilitated by the MAP consumable due to the low thermal mass of each micromolded partition (<1 ng). Using the prototype dPCR platform we were able to show equivalent quantification results with reduced thermal-cycling dwell times. Human genomic DNA from the Coriell repository was used as template along with a FAM-labeled CCL3L1 TaqMan kit and QuantStudio 3D Digital PCR Master Mix v2 (ThermoFisher Cat. A26358; Waltham, MA). The assay and mastermix provider (ThermoFisher) recommended (nominal) thermal-cycling times are 1 cycle of 96°C for 10 minutes followed by 40 cycles of 60°C for 2 minutes and 98°C for 30 seconds. A.) Raw fluoresence results for 100% and 15% nominal (recommended) dwell times. Here the thermal cycling ramp times were not altered, only the hold or dwell times during thermal cycling. Red (ROX channel) represents partitions that filled with reagent but did not contain the template. Green (FAM channel) represents partitions that contained the CCL3L1 template. B.) Quantification results for quadruplicate units at 4 different reduction in dwell times from 100% (control, assay manufacturer recommended dwell times), 50%, 25% and 15%. The error bars show ± 1 standard deviation across the units.

A.

Quadruplicates of 1:50
0.001 pM Takara NGS Standard

Concentration (Cp/µL)	Average (Cp/µL)	C.V. (%)
7.3	7.3	8.6
7.1		
6.6		
8.1		

Quadruplicates of 1:50
0.01 pM Takara NGS Standard

Concentration (Cp/µL)	Average (Cp/µL)	C.V. (%)
84	79.7	8.0
86		
77		
72		

Quadruplicates of 1:50
0.1 pM Takara NGS Standard

Concentration (Cp/µL)	Average (Cp/µL)	C.V. (%)
814	771.5	4.6
787		
742		
743		

Quadruplicates of 1:50
1 pM Takara NGS Standard

Concentration (Cp/µL)	Average (Cp/µL)	C.V. (%)
7960	7720	3.7
7890		
7317		
7713		

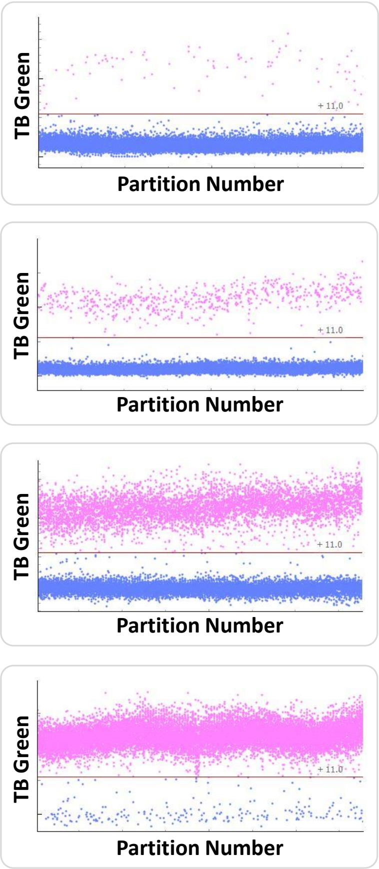

B.

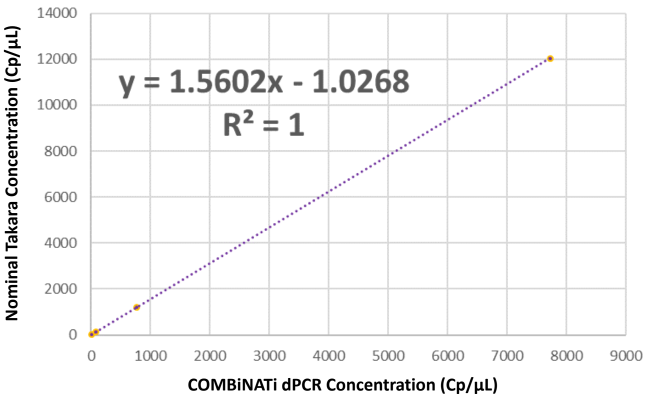

Supplemental Figure 3: *Initial verification of platform function*. PCR reactions were set up using reagents from the Takara NGS Library Quantification Kit (Cat No. 638324). NGS library standards from the kit were diluted 1:50 in the reactions. The consumable was then loaded

and thermal-cycled in the novel dPCR instrument using the parameters suggested by the kit manufacturer. Fluorescence images were collected for both ROX and TB Green (intercalating PCR dye). A) Quadruplicate measurements for 4 orders of magnitude of NGS library standard. The 0.001 pM sample was generated by diluting the supplied 0.01 pM standard 1:10. Scatter plots show one representative unit on the consumable where each point represents the EG Green fluorescence for a single partition. B) Plot of the nominal concentrations provided by Takara versus the average values derived on the COMBiNATi system. The linear regression shows near perfect linearity across the 4 orders of magnitude.

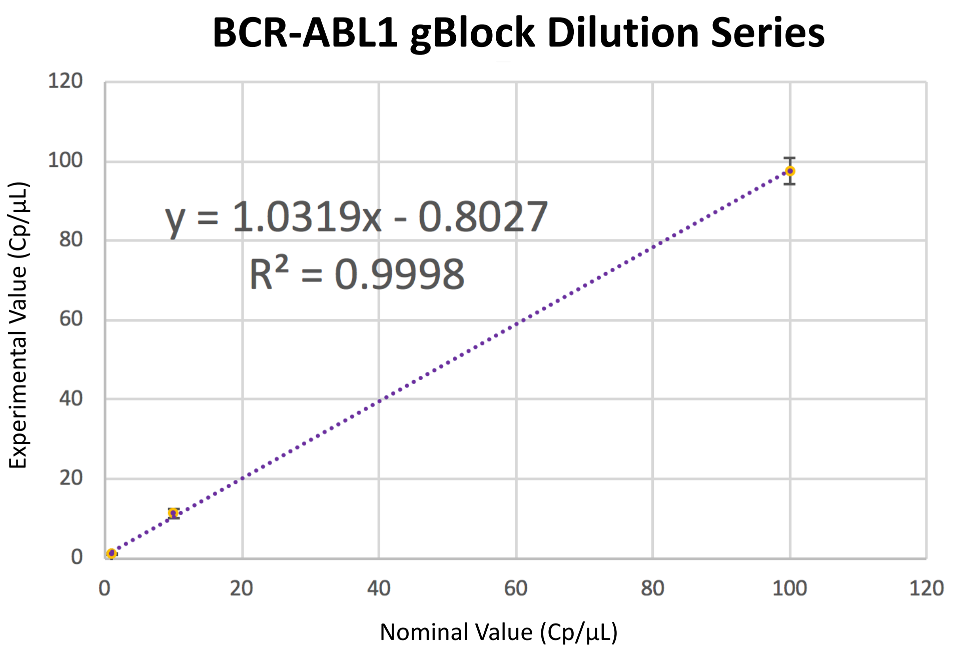

Supplemental Figure 4: *Linearity of BCR-ABL1 Dilution Series.* Using the nominal gBlock concentrations determined prior, PCR reactions were set up to have a 10X dilution series of BCR-ABL1 gBlock template across 3 orders of magnitude and a FAM-labeled BCR-ABL1 probe. This reagent was loaded into a MAP consumable and run on the prototype dPCR instrument. Each point represents the average of triplicate units with y error bars representing standard deviation across the triplicates. The linear regression shows near perfect linearity across the dilutions.
